# Pretreatment of wheat straw leads to structural changes and improved enzymatic hydrolysis

**DOI:** 10.1038/s41598-018-19517-5

**Published:** 2018-01-22

**Authors:** Qi Zheng, Tiantian Zhou, Yibin Wang, Xiaohua Cao, Songqing Wu, Meili Zhao, Haoyuan Wang, Ming Xu, Baodong Zheng, Jingui Zheng, Xiong Guan

**Affiliations:** 1 Engineering and Technology Center of Special Crop Breeding & utilization, Fujian Agricultural and Forestry University, Fujian, Province P.R. China; 2 Key Laboratory of Biopesticide and Chemical Biology, Ministry of Education, Fujian Agriculture and Forestry University, Fuzhou, 350002 P.R. China; 3 College of Food Science, Fujian Agriculture and Forestry University, Fuzhou, 350002 P.R. China

## Abstract

Wheat straw (WS) is a potential biomass for production of monomeric sugars. However, the enzymatic hydrolysis ratio of cellulose in WS is relatively low due to the presence of lignin and hemicellulose. To enhance the enzymatic conversion of WS, we tested the impact of three different pretreatments, e.g. sulfuric acid (H_2_SO_4_), sodium hydroxide (NaOH), and hot water pretreatments to the enzymatic digestions. Among the three pretreatments, the highest cellulose conversion rate was obtained with the 4% NaOH pretreatment at 121 °C (87.2%). In addition, NaOH pretreatment was mainly effective in removing lignin, whereas the H_2_SO_4_ pretreatment efficiently removed hemicellulose. To investigate results of pretreated process for enhancement of enzyme-hydolysis to the WS, we used scanning electron microscopy, X-ray diffraction, and Fourier transform infrared spectroscopy to analyze structural changes of raw and treated materials. The structural analysis indicated that after H_2_SO_4_ and NaOH pretreatments, most of the amorphous cellulose and partial crystalline cellulose were hydrolyzed during enzymatic hydrolysis. The findings of the present study indicate that WS could be ideal materials for production of monomeric sugars with proper pretreatments and effective enzymatic base hydrolysis.

## Introduction

Wheat is one of the major crops grow around the world. Approximately 690 ktons of wheat were produced globally in 2009, later reaching 730 million tons in 2014^1,2^. As the waste residue of wheat, large mass of wheat straw (WS) has been generated every year and become the most abundant agricultural biomass worldwide after rice straw^3^. However, the usual disposal of WS are discard in the field or burned, result in generating issues of great impact on the economic waste and environmental pollution^4,5^. WS is also a major source of renewable energy^6^. As lignocellulosic biomass, WS is inexpensive and abundant, hence, has a great potential for production of bioenergy.

WS is a typical lignocellulosic biomass that mainly comprises cellulose, hemicellulose, and lignin. Cellulose and hemicellulose could be hydrolyzed into monomeric sugars such as glucose, xylose, and arabinose, which could then be converted to biofuels such as bioethanol and methane^7–9^. However, because the chemical components and physical structure of lignocellulosic biomass could protect cellulose from degradation, the bioconversion of these materials has remained challenging^10^. Typically, lignin and hemicellulose in the lignocellulosic materials need to be removed before the enzymatic hydrolysis of cellulose^11^. Various pretreatment approaches have been proposed and applied, including physical, chemical, and biological methods^12^. Alkali, acid, steam explosion, and hot water pretreatments have been extensively performed in related fields^13–15^.

Acids can hydrolyze hemicellulose in lignocellulosic materials. When raw plant materials are treated using acid and high temperature to break strong chemical bonds and to release xylose directly^16,17^, hemicellulose could be broken down to expose cellulose, thereby directly resulting in further degradation by enzymes to produce monomeric sugars such as glucose^18^. For instance, Kim *et al*. treated Jerusalem artichoke with dilute sulfuric acid at concentrations of 0.1 to 8%, followed by autoclaving at 121 °C and 15 psi for 60 min and found that almost all hemicellulose was removed with enhanced enzymatic digestibility, however, the cellulose also decreased when the concentration of H_2_SO_4_ increased^19^.

Difference from acid pretreatment, alkali pretreatment could efficiently remove lignin in plant tissues, leading to high delignification^20,21^. Lignin functions in supporting plant structures to avoid microbial permeation and subsequently deterioration. Moreover, the amorphous heteropolymer is generally insoluble in water^22^. These factors are the main obstacles to the efficient utilization of lignocellulosic biomass. Hence, to enhance enzymatic digestibility, it is necessary to remove lignin from the raw materials. Li *et al*. used four different alkali-based pretreatments to pretreat stems of Jerusalem artichoke and found that alkali pretreatment as the most effective technique in removing non-cellulosic polymers^23^.

Hot water pretreatment is a convenient method that does not cause corrosion. Unlike alkali pretreatments, hot water pretreatment removes the majority of hemicellulose in raw material. However, it is less efficient in removing lignin^24^. Previous studies have revealed that after hot water and acid pretreatment, lignin is deposited as droplets on the surface of solid residues, which inhibited the enzymatic hydrolysis of cellulose in materials^25–29^. Other researchers found that re-localization of lignin during pretreatment improved the accessibility of enzymes to cellulose microfibrils^30–32^.

Methods used for cellulose conversion from WS were investigated previously. For instance, Jaisamut *et al*. (2016) pretreated WS with the combination of 180 °C, 30 min, 1% H_2_SO_4_, and 2.4% Na_2_SO_3_, which resulted in 80% of the cellulose conversion into glucose^2^. Yin and Wang (2017) demonstrated that after irradiation at 100 kGy and 2% NaOH treatment of WS for 1 h, mononeric sugar yield could reach 72.67%^33^. These results indicated that different pretreatment methods could affect composition of WS and might significantly affect yield of sugar production. However, comparison of different concentrations of H_2_SO_4_, NaOH and hot water pretreatment on enzymatic hydrolysis of WS was not been well investigated yet. Furthermore, variations of compositions like cellulose, hemicellulose and lignin contents of WS from different locations were observed^34–36^, Hence, more work is needed to develop better pretreatment approaches for enzymatic hydrolysis of WS. Development of optimized means for conversion of WS in production of sugar may potentially have enormous economic benefits and environment impact. The main objective of present study was to enhance the enzymatic hydrolysis of WS. We tested different concentrations of H_2_SO_4_, NaOH, and hot water pretreatments to the structural changes of WS, followed by treat with enzymes to hydrolyze the cellulose component. Our results suggested that WS could be a potential raw material for producing monomeric sugars.

## Results and Discussion

### Compositions of WS

To investigate the potential resource in WS that could convert to monomeric sugars, the compositions of WS were measured. The composition of WS used in this study, as well as comparison to other WSs, is listed in Table 1. The three types of WSs displayed different compositions, and the contents of cellulose and hemicellulose in WS reported by Constant^35^ (2016) and Merali^36^ (2016) were higher than those of WS in this study. The differences in WS composition may be attributable to variations in geographical location, local temperature, and heterogeneity of feedstock of the WS samples^37^. The cellulose content of raw WS was 33.7% in present study (Table 1), indicating that it is a significant source of carbohydrates that could be further converted to monomeric sugars. However, the high contents of hemicellulose and lignin in WS (19.1% and 19.8%, respectively), which were inhibit the conversion rate of cellulose, leading to the pretreatments of biomass inevitable.

**Table 1:** Composition of raw WS and pretreated WS using various methods: ^a^All of the percentage compositions of raw and pretreated solid samples are calculated based on the dry mass of raw DG; ^b^Conducted at 121 °C for 1 h; ^c^Performed at 175 °C for 1 h; ^d^Data from Constant *et al*. (2016)^35^; ^e^Data from Merali *et al*. (2016)^36^.

Item	Moisture	Ash	Cellulose	Hemicellulose	Lignin
Raw WS^a^ (%)	10.3 ± 0.12	9.7 ± 0.09	33.7 ± 1.62	19.1 ± 1.25	19.8 ± 1.50
2% H_2_SO_4_-pretreated WS^a,b^ (%)			23.9 ± 2.98	4.1 ± 0.22	17.7 ± 1.11
4% H_2_SO_4_-pretreated WS^a,b^ (%)			19.3 ± 0.73	2.4 ± 0.14	15.2 ± 1.17
2% NaOH-pretreated WS^a,b^ (%)			27.1 ± 1.34	13.1 ± 0.89	3.1 ± 0.52
4% NaOH-pretreated WS^a,b^ (%)			24.9 ± 1.09	11.1 ± 1.04	2.2 ± 0.18
Water-pretreated WS^a,b^ (%)			30.2 ± 1.30	17.6 ± 0.89	17.8 ± 0.86
Water-pretreated WS^a,c^ (%)			23.2 ± 0.14	12.3 ± 1.03	16.9 ± 1.17
WS^d^ (%)		3.4	44.3	24.5	
WS^e^ (%)		8.0	37.1	23.5	

### Effect of pretreatments

In order to investigate the effect of different pretreatments, the compositions of WS after pretreatments were determined. Table 1 shows that the residual contents of hemicellulose in WS after 2% and 4% H_2_SO_4_ pretreatments at 121 °C were found to be only 4.1% and 2.4%, respectively, compared to that of 19.1% in the raw WS sample. This finding indicates that acid pretreatment could efficiently remove hemicellulose, which was consistent with the results of a previous study^38^. NaOH pretreatment results in slight degradation of hemicellulose compared to that using H_2_SO_4_ pretreatment (121 °C, 13.1%, and 11.1%, respectively), whereas the contents of lignin after alkali pretreatment were particularly low (121 °C, 4% NaOH, 2.2%), indicating that alkaline solutions efficiently remove lignin by breaking ester bonds, thereby increasing the porosity of biomass^39^. However, cellulose in WS was also degraded during pretreatment. There was only 23.9% and 19.3% remaining cellulose after 2% and 4% H_2_SO_4_ pretreatment at 121 °C. The content of remaining cellulose was higher at 27.1% and 24.9% after 2% and 4% NaOH pretreatment at 121 °C.

Although hot water pretreatment has advantages such as being inexpensive and does not result in corrosion, the degradation of hemicellulose, lignin after hot water pretreatment at 121 °C was not comparable to that using acid and alkali pretreatment (Table 1). The removal of lignin and hemicellulose before enzymatic hydrolysis of lignocellulose is highly necessary because it increases the digestibility of cellulose^9,40^. The low removal efficiency of hemicellulose and lignin by hot water pretreatment at 121 °C indicates that it is not an optimal method for enhancing enzymatic digestibility. Compared to hot water pretreatment at 121 °C, the removal of hemicellulose and lignin was more efficient at 175 °C (12.3% and 16.9%, respectively). However, higher temperatures result in more extensive cellulose degradation.

### Enzymatic hydrolysis

To measure the effect of different pretreatments on cellulose conversation rate, enzymatic hydrolysis was employed. Table 2 shows the conversion rate of cellulose using different pretreatments for the enzymatic hydrolysis of WS. Compared to the cellulose in raw WS, the cellulose conversion rate increased to 49.2% with higher acid concentrations and temperatures. Compared to the cellulose after 4% H_2_SO_4_ pretreatment, the cellulose conversion rate reached to 86.6%. After NaOH pretreatment, the conversion rate of cellulose increased from 38.1% to 65.8% with higher concentrations from 0.5% to 4%. Furthermore, using 4% NaOH, the cellulose conversion rate increased with higher temperature. Compared to the cellulose after NaOH pretreatment, the conversion rate of 2% and 4% NaOH pretreatment was 75.0% and 87.2%, respectively. These high conversion rates could be attributed to the degradation of hemicellulose and lignin after pretreatment (Table 1). Hemicellulose and lignin negatively affects enzymatic hydrolysis by binding to cellulose, thereby impeding its access to cellulose^41,42^. The degradation of hemicellulose and lignin could increase the pore size and accessible surface of the biomass, which in turn enhances accessibility to cellulose, thereby increasing cellulose conversion rate^42,43^. In addition, compared to the cellulose content after pretreatment, the conversion rate of cellulose between 4% H_2_SO_4_ and 4% NaOH was similar (86.6% and 87.2%, respectively). However, as shown in Table 1, during the pretreatments, some of the cellulose was degraded, and the cellulose content after 4% acid pretreatment was lower than that after 4% NaOH pretreatment (19.3% and 24.9%, respectively). Thus, WS pretreated with 4% NaOH could produce higher glucose yield than H_2_SO_4_ pretreatment by enzymatic hydrolysis. This result is consistent with the previous study^17^. However, the cellulose conversion rate reach to 100% at 4% NaOH previously^17^, whereas only 87.2% in present study. This may be attributable to distinct biomass saccharification of different lignocellulosic materials. The cellulose conversion rate of hot water pretreatment at 121 °C was only 28.3%. Furthermore, compared to the cellulose in raw and pretreated WS, 32.9% and 48.7% cellulose was converted to glucose after hot water pretreatment at 175 °C, respectively, which is significantly low compared to that using NaOH pretreatment.

**Table 2:** Wheat straw pretreatment conditions and the cellulose conversion rates: statistical significance is indicted with superscript asterisk: ^*^P < 0.05, ^**^P < 0.01, ^***^P < 0.001, ^****^P < 0.0001, and ns indicted not statistically different at *P* = 0.05; ^a^Compared to cellulose in raw WS; ^b^Compared to cellulose in pretreated WS.

Sample no.	Temp. (°C)	Conversion rate of cellulose^a^ (%)	Conversion rate of cellulose^b^ (%)
0.5% H_2_SO_4_-pretreated	121	36.3 ± 1.84^****^	
1% H_2_SO_4_-pretreated	121	39.8 ± 1.55^****^	
2% H_2_SO_4_-pretreated	121	45.9 ± 1.33^****^	65.6 ± 1.90^****^
4% H_2_SO_4_-pretreated	121	49.2 ± 0.42^****^	86.6 ± 0.73^ns^
4% H_2_SO_4_-pretreated	50	19.2 ± 0.49^****^	
4% H_2_SO_4_-pretreated	100	23.1 ± 0.75^****^	
0.5% NaOH-pretreated	121	38.1 ± 1.66^****^	
1% NaOH-pretreated	121	56.4 ± 3.47^***^	
2% NaOH-pretreated	121	59.7 ± 0.96^ns^	75.0 ± 1.21^****^
4% NaOH-pretreated	121	65.8 ± 0.17	87.2 ± 3.42
4% NaOH-pretreated	50	55.6 ± 0.44^****^	
4% NaOH-pretreated	100	59.3 ± 2.30^*^	
Water-pretreated	121	28.3 ± 1.05^****^	28.7 ± 1.07^****^
Water-pretreated	50	21.8 ± 1.04^****^	
Water-pretreated	100	29.7 ± 1.56^****^	
Water-pretreated	175	32.9 ± 1.41^****^	48.7 ± 1.78^****^

### SEM observation

**Figure 1:**
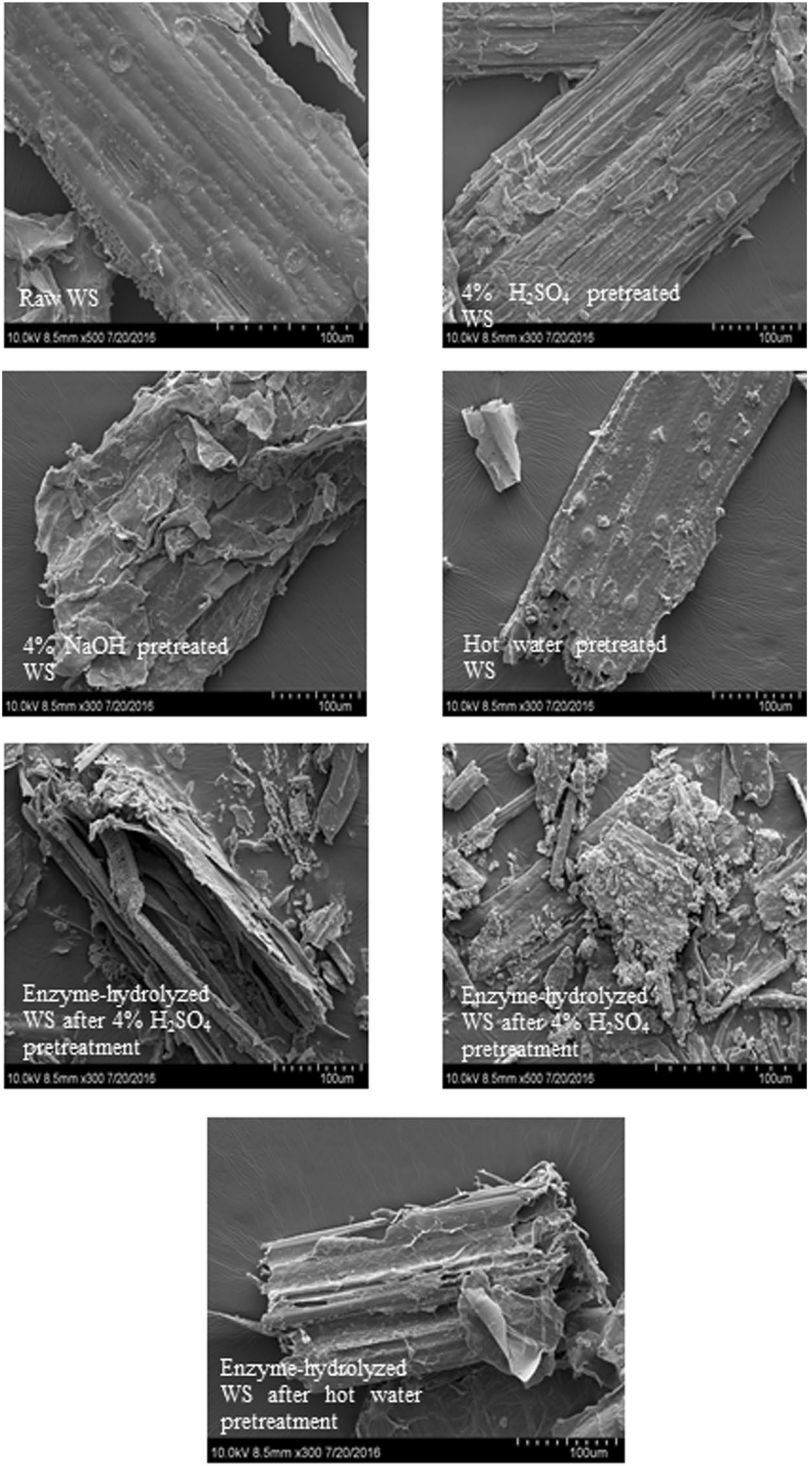
SEM images: all samples were exposed to 121 °C for 1 h except for raw WS.

To observe the structure changes of the WS, SEM analysis was conducted. The morphological features of raw, pretreated, and enzyme-hydrolyzed WS are shown in Figure 1. Raw WS showed a regular and compact surface structure with fibers arranged in bundles, which impede cellulose access by cellulase. The surface of the WS samples, which is mainly composed of lignin and hemicellulose, was destroyed after acid and alkali pretreatment (Figure 1). The lignin and hemicellulose of the pretreated WS samples were partially removed and broken or became loose, thereby resulting in the exposure of internal structures. Especially after NaOH pretreatment, the WS became loose and scattered, and exhibited fiber porosity on its surface compared to the raw WS. These findings demonstrate that pretreatment could destroy the cellulose-hemicellulose-lignin network, thereby removing some of the external fibers^9^. The exposure of internal structures of the WS samples increases the accessibility of cellulase to the inner cellulose, thereby accelerating the biodegradation process. However, hot water pretreatment only caused minimal changes on the surface of the WS samples (Figure 1), which is consistent with the low efficiency removal of hemicellulose and lignin (Table 1). The structures of the enzymatically hydrolyzed WS samples after acid- and alkali-pretreatments were completely destroyed, indicating that most of the cellulose was degraded by the enzyme. The surface of the enzymatically hydrolyzed WS samples after hot water pretreatment was relatively smooth and showed minimal destruction compared to that using acid and alkali pretreatment, which is indicative of the inefficient removal of hemicellulose/lignin and low enzymatic digestibility of cellulose in WS.

### XRD analysis

**Figure 2:**
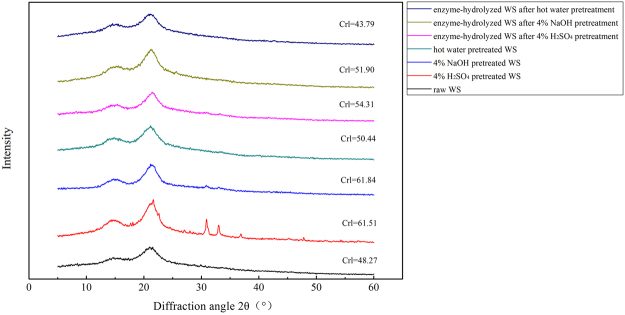
XRD patterns: all samples were exposed to 121 °C for 1 h except for raw WS.

Due to crystalline feature of cellulose, pretreatment could potentially enhance the diffusion of cellulase through amorphous cellulose and markedly affect the enzymatic accessibility of lignocellulosic biomass^44,45^. The crystallinity index (CrI) is an important indicator closely related to enzymatic digestibility because it could reflect biomass crystallinity. It could also indirectly indicate the amorphous phase signal of the biomass such as lignin, hemicellulose, and cellulose domains^38,46,47^. The CrI of raw, pretreated, and enzyme-hydrolyzed WS is shown in Figure 2. Compared to the CrI of raw WS (48.27%), the CrI of acid-pretreated and alkali-pretreated WS was considerably higher (61.51% for acid-pretreated WS and 61.84% for alkali-pretreated WS, respectively). The conversion rates of these two pretreatments were as high as 86.6% and 87.2%, which were calculate on cellulose content after pretreatment. This could be attributed to the fact that hemicellulose and lignin were partially degraded during the pretreatment (Table 1). Thus, the crystallinity of the biomass increased, thereby leading to a high cellulose conversation rate. Similar result was reported by previous studies. Haque *et al*.^48^ reported that compared to raw WS (45.0%), the CrI of WS samples increased to 52.3%, 57.9%, 65.3%, and 71.5% after pretreatment with 0.5%, 1.0%, 1.5%, and 2.0% NaOH at 105 °C, and the reducing sugar yield increased from 45% to 78% with higher NaOH concentrations (1%, 1.5%, and 2.0%) and CrIs. The CrI of hot water-pretreated WS (50.4%) slightly increased compared to that of raw WS, and the conversion rate of cellulose was only to be 28.3%. This could be explained by the inefficient removal of hemicellulose and lignin. The CrIs of enzyme-hydrolyzed WS samples decreased compared to those of pretreated WS samples, which may be attributable to the partial degradation of crystalline cellulose.

### FTIR characterization

**Figure 3:**
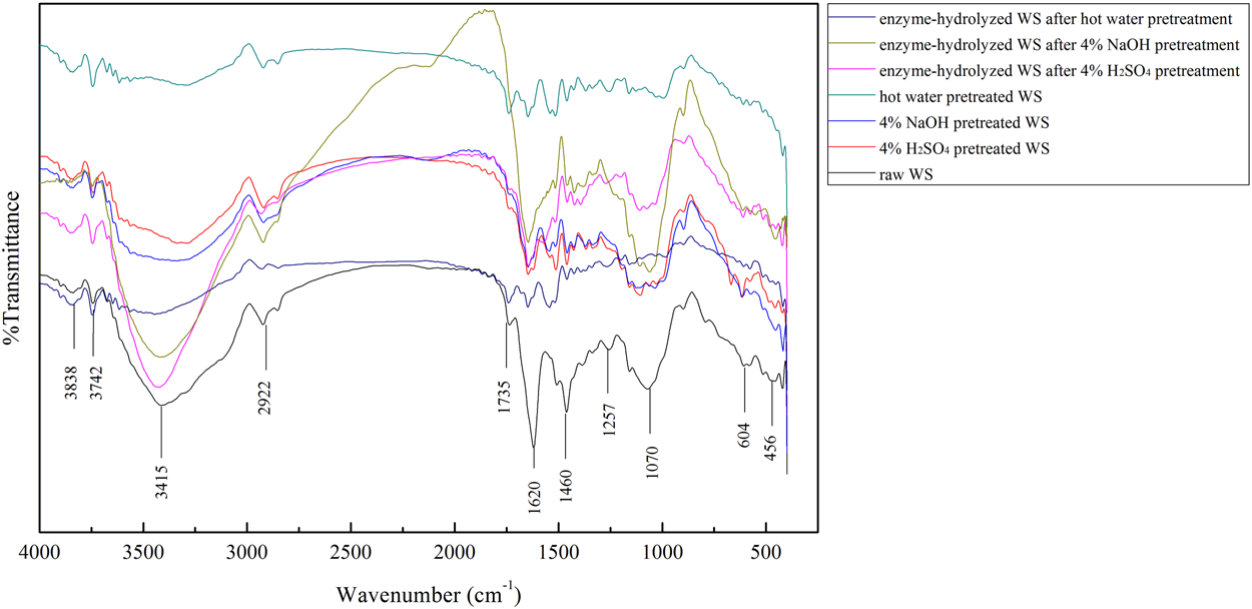
FTIR spectra: all samples were exposed 121 °C for 1 h except for raw WS.

The FTIR spectra of raw WS, pretreated WS, and enzyme-hydrolyzed WS are shown in Figure 3. The broad band ranging from 3,000–3,500 cm^−1^ was assigned to the O-H stretching vibration^9^. The intensity of these absorption peaks indicate the cellulose content in the WS samples^38^. The intensities of these bands in the FTIR spectra of 4% H_2_SO_4_, 4% NaOH, and hot water pretreatments were significantly higher than those of enzyme-hydrolyzed WS. These results suggest that the cellulose content of pretreated WS was higher than those of enzyme-hydrolyzed WS. The bands around 2,850–2,922 cm^−1^ were attributed to the C-H stretching vibration of the aliphatic chain structure of lignin^49^. The bands of 4% H_2_SO_4_ pretreatment, hot water pretreatment, and enzyme-hydrolyzed WS after 4% hot water pretreatment were similar to that of raw WS, indicating weak lignin removal. Compared to the raw WS, the intensities of these bands in the spectra after NaOH pretreatment and enzyme-hydrolyzed WS after NaOH pretreatment significantly decreased, which indicates that the aliphatic compounds in lignin were efficiently removed. These results are related to the fact that alkali pretreatment was more efficient in removing lignin. Furthermore, compared to the raw WS, the bands ranging from 1,635–1,655 cm^−1^ disappeared, which were attributed to the removal of hemicellulose^50^. The range of 500–1,770 cm^−1^ is considered as the lignin fingerprint region^9^. The intensities of the absorption peaks at 1,735, 1,620, 1,460, 1,257, 1,070, and 604 cm^−1^ decreased or disappeared compared those of raw WS, indicating that lignin and hemicellulose in the pretreated WS samples were partially removed^9^. The bands within the range of 1,450–1,630 cm^−1^ were attributed to the aromatic skeleton stretching vibration, and the intensities of these absorption peaks were stronger than those of raw WS^38^.

In conclusion, the effects of H_2_SO_4_, NaOH, and hot water pretreatment on cellulose digestibility of WS were investigated. Our findings indicate that H_2_SO_4_ pretreatment could efficiently remove hemicellulose in WS. However, the removal of lignin was poor and only partial cellulose degradation was observed with H_2_SO_4_ pretreatment compared with previous study^19^. The cellulose conversion rate was as high as 86.6% with 4% H_2_SO_4_ pretreatment (121 °C). NaOH pretreatment could efficiently remove lignin and partially degrade cellulose in WS. Furthermore, among all the pretreatments, the highest conversion rate was obtained by 4% NaOH pretreatment at 121 °C (87.2%), which was pretreated at a mild alkali concentration and temperature. Although slight cellulose degradation was observed with hot water pretreatment, the removal of hemicellulose and lignin were not comparable to that using acid and alkali pretreatment, and the cellulose conversion rate by enzymatic hydrolysis was particularly low (28.3%). SEM analysis revealed that lignin and hemicellulose in WS were destroyed after acid and alkali pretreatment, whereas hot water pretreatment only caused minimal changes on the surface of the WS samples. The CrIs of cellulose increased after pretreatments, particularly that using H_2_SO_4_ and NaOH pretreatment, which resulted in an enhancement of enzymatic hydrolysis. Thus, NaOH pretreatment at a mild concentration and temperature (4% v/v, 121 °C for 1 h) was determined to be the optimal method for enhancing the enzymatic digestibility of WS.

## Materials and Methods

### Materials

The wheat straw was collected from a farm located in Deyang, Sichuan, China in June 2016. After drying at 45 °C in an oven for two weeks, the WS was milled with a Wiley laboratory mill (Model No. 4, Thomas Scientific, Philadelphia, PA, USA), followed by passing through a 40-mesh screen. The obtained samples were stored in airtight plastic bags for the following experiments. The moisture content and ash content of the samples were determined according to the National Standard Procedures of People’s Republic of China^51,52^. To determine moisture content, 1–2 g of the WS was placed in a preweighed weighing bottle and dried at 105 °C for 4 h. The weighing bottle was then transferred to a desiccator, cooled for 0.5 h, and then weighed. The weighing bottle was again moved, dried for 1 h, and cooled for weighing until constant weight. The moisture content was calculated based on the National Standard Procedures of People’s Republic of China (Eq. 1)^52^. For ash content, 2–3 g of WS was placed in a preweighed porcelain crucible and carbonized in a muffle furnace at 225 °C, followed by burning to ash at 575 °C. The crucible was air cooled for 1 min and then cooled in a desiccator for 0.5 h and weighed. The porcelain crucible was dried at 105 °C and cooled for weighing until constant weight. Ash content was calculated on based on the National Standard Procedures of People’s Republic of China (Eq. 2)^51^. Cellulose and hemicellulose of the samples was determined by the method of the National Renewable Energy Laboratory (NREL)^53^. Briefly, 0.5 g of WS was placed in the qualitative filter paper that was soaked in ethanol for 1–2 h and extraction was performed for 16–24 h in a Soxhlet extractor. After drying, 3 mL of 72% H_2_SO_4_ was added and incubated for 1 h at 30 °C. Approximately 84 mL of deionized water was added to dilute the acid to 4%, followed by heating at 121 °C for 45 min. The solution was filtered through a Buchner funnel, and the filtrate was collected into a new Erlenmeyer flask. The liquid was neutralized using CaCO_3_ and stored at −20 °C for the determination of cellulose and hemicellulose content using HPLC. The lignin content was determined using the method described by Chung *et al*.^54^. 5 mg of sample was suspended in 2.5 mL acetyl bromide (25% AcBr in acetic acid). 0.1 mL of perchloric acid was added and mixed. The mixture was dried three times in an oven for 10 min each at 70 °C and cooled on ice water for 30 min, followed by transferred to volumetric flask containing 10 mL of 2 M NaOH and 12 mL of acetic acid. Lignin content was determined by a UV-VIS at 280 nm using 20.09 g^−1^·L·cm^−1^. All composition percentages of the raw and pretreated solid samples were calculated on dry mass of WS.

1 $${\rm{Moisture}}\,{\rm{content}}\,( \% )=({m}-{{m}}_{{\rm{1}}})/{m}\times 100;$$

where *m* is the weight of WS before being dried, g; and *m*_1_ is the oven dry weight of WS, g.

2 $${\rm{Ash}}\,{\rm{content}}\,( \% )=({{m}}_{2}-{{m}}_{1})/{m}\times 100;$$

where *m*_*1*_ is the weight of crucible, g; *m*_2_ is the weight of crucible with ash, g; and *m* is the oven dry weight of WS, g.

### Pretreatments of WS

Three different pretreatments were tested in this study, including sulfuric acid pretreatment (0.5–4% w/v, 50–121 °C, 1 h), sodium hydroxide pretreatment (0.5–4% w/v, 50–121 °C, 1 h), and hot water pretreatment (50–175 °C, 1 h). The pretreatment was performed according to a previous study^41^. Briefly, 5 g of WS was placed in 50 mL of pretreatment liquid and sealed in an Erlenmeyer flask in an autoclave and then heated at different temperatures for 1 h (Table 2). For hot water pretreatment at 175 °C, 5 g of WS was placed in 50 mL deionized water and sealed in a stainless steel pot, followed by heating in an oil bath digester (YYQ-10-1.25, Nanjing Jiezhen Science & Technology Development Co. Ltd., Nanjing City, China) for 1 h. Each sample was immediately cooled and neutralized using HCl or NaOH heat treatment. The neutralized sample was centrifuged (7,000 *g*) at 4 °C for 3 min. The residue was washed four times with 100 mL of deionized water and centrifuged previously descried, followed by dried at 45 °C for enzymatic hydrolysis.

### Enzymatic hydrolysis of pretreated WS

Enzymatic hydrolysis of the pretreated WS as described in a previous study^55^. Briefly, 1 g of the pretreated WS was immersed in 30 mL of a sodium citrate buffer (pH 4.8, 50 mM). To hydrolyze the lignocellulose biomass, 35 FPU/g cellulase from *Trichoderma reesei* (Celluclast 1.5 L) and 61.5 CBU/g cellobiase from *Aspergillus niger* (Novozym 188) were added. In addition, sodium azide (0.3% w/v) was also added to the solution to prevent microbial contamination. The lignocellulose biomass was dynamically (150 rpm) incubated at 55 °C for 72 h and then rapidly cooled on ice to stop the reaction. The hydrolysate was then centrifuged to collect the supernatant for the determination the yield of monomeric sugar using HPLC.

### SEM observation

The morphologies of raw, pretreated, and enzyme-hydrolyzed WS were characterized by SEM (SU8010, Hitachi Hightechbologies Co., Tokyo, Japan) as described elsewhere^56^. All samples were conducted at a 4% concentration and 121 °C for 1 h except for raw WS. Before SEM observation, a thin layer of gold was sputter-coated on the samples to prevent degradation and to render fiber conductivity.

### XRD analysis

XRD (Ultima UVX XRD, Rigaku Co., Tokyo, Japan) was used to characterize the crystalline phases of raw, pretreated, and enzyme-hydrolyzed WS, which was operating at 30 kV and 10 mA with Cu radiation (1.54 Å) as previously described^57^. Except for raw WS, all samples were conducted at a 4% concentration and 121 °C for 1 h. The X-ray diffractograms were recorded from 10° to 40° with a step size of 0.02°. The CrI was calculated using the following Eq. (3)^58^.

3 $$CrI\,( \% )=({I}_{{002}}-{I}_{am})/{I}_{002}\times 100 \% ;$$

where *I*_002_ is the maximum peak intensity at lattice diffraction; and *I*_*am*_ is the intensity of diffraction at 2θ = 18°.

### FTIR analysis

FTIR analysis was performed to reveal the changes in the functional groups in raw, 121 °C pretreated and enzyme-hydrolyzed WS^47^. The FTIR spectra of different samples were obtained with a FTIR spectrometer (Nicolet 8700, Bruker AXS, Karlsruhe, Germany). The data was recorded within a range of 400–4,000 cm^−1^ with a detector at 4 cm^−1^ resolution and 128 scans per sample.

### Analytical methods

The yields of monomeric sugars were measured using HPLC. Precolumn derivatization with PMP was performed with an Agilent Zorbax SB-C18 column^59^. The experiments were conducted in triplicate. All data were expressed as the mean ± standard deviation and analyzed with GraphPad Prism 6 via the Turkey−Kramer test. The cellulose conversion rate was calculated as Eq (4).

4 $${\rm{Cellulose}}\,{\rm{conversion}}\,{\rm{rate}}\,( \% )={{C}}_{{glucose}}\times 0.9/{{C}}_{{cellulose}}\times 100 \% ;$$

where *C*_*glucose*_ is the concentration of glucose in enzymatic hydrolysate, g/L; and *C*_*cellulose*_ is the concentration of cellulose in samples, g/L.
